# Genome Sequence of a Newly Isolated F2 Subcluster Mycobacteriophage from the Black Belt Geological Region of Western Alabama

**DOI:** 10.1128/genomeA.01555-17

**Published:** 2018-01-25

**Authors:** Kayla M. Fast, Tracy Keener, Rakim Ali, Brittany M. Butcher, Joshua D. Millwood, Timothy Odom, P. Kiersten Schellhammer, Ezekiel Ufomadu, Michael W. Sandel

**Affiliations:** a Department of Biological and Environmental Sciences, University of West Alabama, Livingston, Alabama, USA

## Abstract

The bacteriophage Demsculpinboyz was discovered in a soil sample from the Black Belt region of Alabama using *Mycobacterium smegmatis* mc^2^155 as its host. The genome is 57,437 bp long and contains 116 protein-coding genes. It belongs to the F2 subcluster, which has only five other members.

## GENOME ANNOUNCEMENT

Actinobacteriophages are an excellent model for pursuing evolutionary questions because of the extreme amount of genetic diversity present across phage genomes (1). Additionally, phage genomes are considerably old and dynamic due to genome mosaicism and horizontal gene transfer (2, 3). Phage particles are the most abundant biological entities in the environment and can be isolated from soil with relative ease (4). Students and faculty at the University of West Alabama (UWA) have participated in the Science Education Alliance-Phage Hunters Advancing Genomics and Evolutionary Science (SEA-PHAGES) program and have contributed to the growing wealth of phage genomes discovered by undergraduate students (5).

The mycobacteriophage Demsculpinboyz was discovered in soil from an orchard in Livingston, AL (32.594337 N, 88.186615 W). This phage was isolated from the bacterial host (*Mycobacterium smegmatis* mc^2^155) and cultured in 7H9 medium. Following phage purification and amplification, the genome was sequenced by using an Illumina MiSeq instrument with 1,425× shotgun coverage. Trimmed reads were assembled by using Newbler, yielding a 57,437-bp contig with a G+C content of 60.9%. Genome ends were apparent from a buildup of reads beginning on a single base, and a 10-bp 3′ complementary single-stranded DNA (ssDNA) extension was identified between the ends. Transmission electron microscopy showed that Demsculpinboyz belongs to the *Siphoviridae* family and has a short tail with an icosahedral capsid.

Protein-coding genes in the genome were autoannotated and identified using Glimmer and GeneMark, proceeded by manual inspection and annotation editing. A total of 116 protein-coding genes were predicted, making up 94.75% of the genome coding capacity. This genome contains no tRNAs. BLASTn analysis showed that Demsculpinboyz is closely related to the mycobacteriophages Avani, Che9d, and Zapner. The closest relative is Che9d, which shares 98% nucleotide identity across 72% of the genome. There are multiple syntenic interruptions from sequence differences potentially due to insertions, deletions, or gene substitutions.

Demsculpinboyz belongs to mycobacteriophage subcluster F2. The F2 subcluster represents a relatively small proportion of phages within the F cluster, containing only six phages. The mean genome size within the F2 subcluster is 56,236 bp, and the average gene number is 111. Demsculpinboyz and other F2 phages show high nucleotide similarity and share a conserved region in the first third of the genome. A Phamerator map of Che9d and Demsculpinboyz shows a decrease in nucleotide similarity at 30,000 bp (6).

Within the F2 subcluster, the Lysin A gene (Demsculpinboyz gp35) is represented by three variations of conserved domains. In mycobacteriophages, Lysin A is modular and typically composed of a C-terminal domain, a central catalytic domain, and an N-terminal domain (7). Lysin A is an endolysin essential for host cell lysis during the lytic cycle. Domain organization in Lysin A is well conserved, but few clusters include multiple modules as F2 does (7). All F2 phages, at the minimum, are composed of a central amidase-2 domain variant (Ami-2A or Ami-2B) and a C2 C-terminal domain (7). The two phage sequences with highest overall similarity to Demsculpinboyz (Avani and Che9d) lack an N-terminal domain and contain a second central domain, GH19 (7).

### Accession number(s).

The Demsculpinboyz genome sequence has been deposited in GenBank under the accession no. MF919502.
